# Selection and drift reduce genetic variation for milk yield in Manech Tête Rousse dairy sheep

**DOI:** 10.3168/jdsc.2020-0010

**Published:** 2020-12-11

**Authors:** Fernando L. Macedo, Ole F. Christensen, Andrés Legarra

**Affiliations:** 1 GenPhySE, INRAE, 31326 Castanet Tolosan, France; 2 Facultad de Veterinaria, UdelaR, A. Lasplaces 1620, Montevideo, Uruguay; 3 Center for Quantitative Genetics and Genomics, Blichers Allé 20, 8830 Tjele, Denmark

## Abstract

Decreases in genetic variance over generations reduce future genetic gain. We studied the evolution of genetic variance in the dairy sheep breed Manech Tête Rousse, which has been selected for increasingly complex objectives, including, in this order, milk yield, milk contents, scrapie resistance, and somatic cell score. We estimated base population genetic variance and genetic variance by sex and per year of birth from 1981 to 2014. The data consisted of 1,842,295 milk yield records (from 1978 to 2017) and a pedigree including 530,572 females (96% of them with records) and 3,798 artificial insemination males. As a measure of drift, we computed average relationships for each cohort from which we derived expected reduction of variance due to increased relationships. The difference between observed and expected reductions in genetic variances is the reduction in genetic variance due to selection. Average relationships increased steadily but slowly in both sexes. For females, genetic variance reduced with time until a plateau was reached at around 90% of the initial genetic variance. The reduction due to relationships (roughly 3% cumulated in 30 yr) was smaller than that due to selection (roughly 10% across the last years). A smaller loss due to selection was seen in recent years, possibly due to a change in selection objectives. These results agree well with theoretical expectations. The pattern of the evolution of genetic variance in males was similar to that for females but with a stronger reduction because of strong selection of AI males at birth. We conclude that the reductions in genetic variation due to selection and drift agree with expectations, and none of the reductions are very strong in this population because of control of inbreeding and smooth changes in selection objectives over time.

## Highlights

• Genetic variance is important to estimate future genetic progress
• Selection and drift reduce genetic variance
• In Manech Tête Rousse, the loss of genetic variance was about 13% for females
• Bulmer effect had a greater influence (10%) than drift (3%)
• Changes in breeding objectives affect the evolution of genetic variance

## Graphical Abstract

**Figure:**
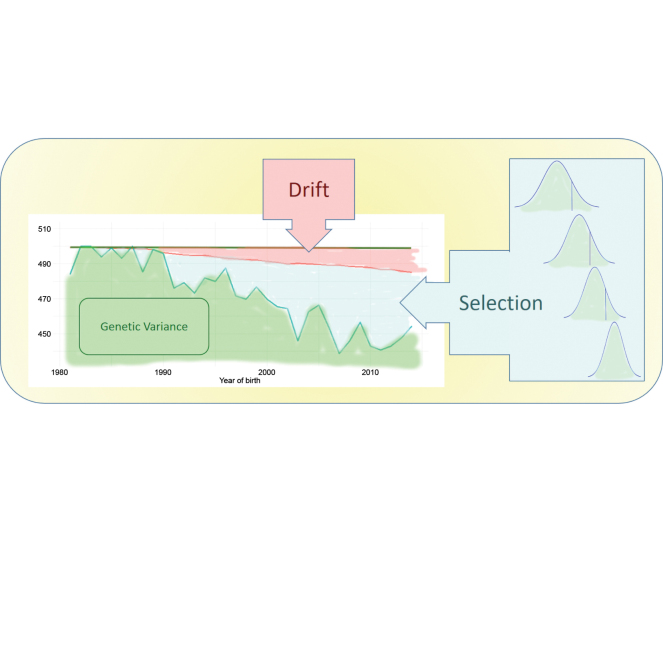
**Summary:** Genetic variance is one of the bases for selection. Drift and selection reduce genetic variance and therefore reduce the expected genetic gain. Few works with actual data have analyzed the effect of selection over the genetic variance. Moreover, these works do not distinguish between drift and selection effects. In our study, we analyzed the effect of drift and selection over the loss of genetic variance for milk yield for a period of 33 years in a population of French dairy sheep Manech Tete Rousse. We observed a stabilization at 13% of loss of genetic variance, with 3% due to drift and 10% due to selection. In conclusion, neither drift nor selection has an important effect on the loss of the genetic variance of milk yield in this population.

There are 2 processes in the evolution of genetic variance under artificial selection. First, there is an effect of limited population size, by which the buildup of coancestry (half the relationship coefficient) reduces genetic variation as animals become increasingly related. This reduction is well known and can be understood as drift (Sorensen and Kennedy, 1984; Falconer and Mackay, 1996). It acts independently of selection; that is, it is only due to demographic factors and is the same for all traits. Second, selection causes directed changes in allele frequencies and negative linkage disequilibrium (**LD**) among QTL, also known as the Bulmer effect. In an infinitesimal model and in the short term, the reduction of genetic variance is due mostly to negative covariance between QTL, whereas directed changes in allele frequencies have a small impact (Bulmer, 1971; Walsh and Lynch, 2018, Chapters 11 and 16). However, the reduction of genetic variation due to LD is not constant, with a significant reduction until the third or fourth generation, when it becomes stable, as there is an equilibrium between recombination and LD (Dekkers, 1992; Villanueva et al., 1993). Typical values of reduction of genetic variance due to the Bulmer effect, until its stability, range from 5 to 20%. Genetic variance of the unselected population is “genic” variance, whereas genetic variance of the population at hand for selection (eventually, after reduction due to selection) is “genetic” variance (Walsh and Lynch, 2018, Chapters 11 and 16).

Reduction of genetic variance affects genetic gain and its prediction. The genetic gain is often predicted based on base population parameters, without accounting for the Bulmer effect. The reduction in genetic variance also decreases heritability, thus affecting accuracy of selection (Bulmer, 1971; Dekkers, 1992; Bijma, 2012; Gorjanc et al., 2015). The Bulmer effect is well understood in simplified contexts based on selection index theory (Dekkers, 1992; Villanueva et al., 1993; Rutten et al., 2002). Villanueva et al. (1993) quantified that between 8% and 26% of the reduction in the response of multivariate BLUP selection was due to the Bulmer effect.

Recent introduction of genomic selection has renewed interest in the Bulmer effect (van Grevenhof et al., 2012; Gorjanc et al., 2015; Allier et al., 2019; Hidalgo et al., 2020). van Grevenhof et al. (2012) showed by deterministic simulations that the decrease in genetic gain is the same for genomic selection and for traditional BLUP selection. However, there are very few estimates of the reduction of genetic variance in selected populations based on actual records. Allier et al. (2019) estimated that the Bulmer effect accounted for a 23% reduction in genetic variance. They compared the genetic variance in the existing selected population of maize, which is in LD, to the genetic variance in a hypothetical population in linkage equilibrium. However, in their study, there is no base population in a pedigree sense; that is an ancestral, unselected population. Hidalgo et al. (2020) reported substantial reductions of genetic variance in a pig population selected for growth and fitness traits. However, neither Allier et al. (2019) not Hidalgo et al. (2020) decomposed the reduction in genetic variance into the loss of genetic diversity due to drift (i.e., the buildup of coancestry among individuals) and the Bulmer effect (i.e., the buildup of LD across QTL). To prepare optimal strategies for long-term breeding, it would be of interest to disentangle these 2 phenomena; for instance, the Bulmer effect is smaller when the selection objective changes or when the next generation is produced by mating at random, whereas the loss due to coancestry could be handled by strategies such as optimal contribution selection (Woolliams et al., 2015).

Dairy sheep is an interesting species in which to study the Bulmer effect. In France, the cooperative schemes have strategies at the breed level to handle inbreeding. These schemes also have clearly defined and consensual selection objectives at each time period. This is opposite to dairy cattle where different AI studs may propose different portfolios of bulls, and breeding objectives and strategies may differ among actors. Also, in dairy sheep, the populations are large enough (tens of thousands of animals born yearly) for accurate inferences of the evolution of Bulmer effect. The objective of this work was to estimate the trajectory of genetic variance over years, and the reduction of genetic variance due to coancestry and Bulmer effect, for the trait yearly milk yield in Manech Tête Rousse (**MTR**) sheep, a breed that extensively uses AI (>70% of replacement females are born from AI).

We used all available milk yield records (from 1978 to 2017) and pedigree of MTR (roughly with the same time span as the milk records). A total of 1,842,295 records of milk yield and 540,999 individuals were included in the pedigree (530,572 females, of which 96% have records, and 3,798 AI males). The average generation interval was approximately 4 yr, so there are about 10 generations. There is no formal use of optimal contribution selection in this population, but matings among cousins are avoided, so recent inbreeding is avoided. Data were precorrected for heterogeneity of variances using the method of Meuwissen et al. (1996) to avoid scale effects. Breeding objectives in MTR are relevant for interpretation of the results. From the start of the breeding program in the 1980s until 2003, the only objective was milk yield per annual lactation. From 2003 until 2016, the breeding objective was fat and protein yields (genetically highly correlated with milk yield), which gradually changed to fat and protein contents, to prevent deterioration of cheese-making (Barillet, 1997). From 2016, SCS was added. From 2000 to 2005, there was also an emphasis in selecting scrapie-resistant rams (Palhiere et al., 2008), which partly diminished selection pressure on other traits.

Genetic evaluation was by pedigree BLUP animal model with permanent environment effect to account for repeated measurements. The linear model included contemporary group (flock, year, and lactation number), age, lactation number, month of lambing, and interval lambing to first milk recording. Random effects were animal and permanent environment. Because there are approximately 20% missing sires in the pedigree, the model included 13 unknown parent groups every 3 yr.

We followed the method presented in Sorensen et al. (2001). We focused on the evolution of male (AI rams) and female (commercial females at farms) genetic variances along time, although the method is very general and can be applied for any partition of animals of interest. We used Gibbs sampling with 150,000 iterations, a burn-in of 15,000, and saving samples each 150 iterations. We obtained the posterior distribution of the genetic variance at the base population, $\sigma_{a}^{2}$. Also, at each 150th iteration, we took samples of EBV for groups of individuals formed by sex (males and females) and year of birth year *t* (1981 to 2014). We computed the variance of the samples of EBV for each of these 34 × 2 = 68 groups. These variances were, in turn, samples from the posterior distributions of genetic variances of males (*m*) at time *t* $\left(\sigma_{a\left(m\right)}^{2\left(t\right)}\right)$ and genetic variance of females (*f*) at time *t* $\left(\sigma_{a\left(f\right)}^{2\left(t\right)}\right)$. Thus, at the end of the process, we had the posterior distribution (with 900 samples) of the genetic variance for each of the 34 groups of AI males ${\hat{\sigma }}_{a\left(m\right)}^{2\left(t\right)}$, each of the 34 groups of females ${\hat{\sigma }}_{a\left(f\right)}^{2\left(t\right)}$, and of the genetic variance in the base population $\left({\hat{\sigma }}_{a}^{2}\right)$.

The expected genetic variance as a function of average inbreeding $\left({\bar{F}}_{t}\right)$ and the average relationship ( ${\bar{\text{A}}}_{t}$, where ${\text{A}}_{t}$ is the corresponding submatrix of additive relationships) of animals born at time *t* is

$$E\left(\sigma_{a}^{2\left(t\right)}\right)=\sigma_{a}^{2}\left(\bar{diag\left({\text{A}}_{t}\right)}-{\bar{\text{A}}}_{t}\right)=\sigma_{a}^{2}\left(1+{\bar{F}}_{t}-{\bar{\text{A}}}_{t}\right)$$

(Sorensen et al., 2001; Legarra, 2016). This expression considers that animals at time *t* are inbred (which increases the variance) and related (which decreases the variance). The reasoning extends to separate sexes by computing separate averages ${\bar{F}}_{t}$ and ${\bar{\text{A}}}_{t}$. Relationships and inbreeding were obtained using INBUPGF90 (Aguilar and Misztal, 2008).

The difference between the observed genetic variance ${\hat{\sigma }}_{a}^{2\left(t\right)}$ and expected genetic variance $E\left(\sigma_{a}^{2\left(t\right)}\right)$ is considered to be the reduction of genetic variance due to selection. This includes both the Bulmer effect and the preselection of animals at birth based on parent average. Preselection is strong in males but mild in females. We assumed that the genetic variance in females is representative of the population. The expected reduction due to increased relationships was $\sigma_{a}^{2}-\sigma_{a}^{2}\left(1+{\bar{F}}_{t}-{\bar{\text{A}}}_{t}\right)=\sigma_{a}^{2}\left(-{\bar{F}}_{t}+{\bar{\text{A}}}_{t}\right)$. The observed reduction in genetic variance was $\sigma_{a}^{2}-{\hat{\sigma }}_{a}^{2\left(t\right)}$. Thus, the reduction due to Bulmer and preselection was $\left(\sigma_{a}^{2}-{\hat{\sigma }}_{a}^{2\left(t\right)}\right)-\left[\sigma_{a}^{2}-\sigma_{a}^{2}\left(1+{\bar{F}}_{t}-{\bar{\text{A}}}_{t}\right)\right]=\sigma_{a}^{2}\left(1+{\bar{F}}_{t}-{\bar{\text{A}}}_{t}\right)-{\hat{\sigma }}_{a}^{2\left(t\right)}$, the expected minus the observed genetic variance. Again, this reasoning extends easily to separate sexes. For example, if $\sigma_{a}^{2}$ in the base population is 100, and $1+{\bar{F}}_{f,2000}-{\bar{\text{A}}}_{f,2000}=0.9$, the expected genetic variance in females in year 2000 is $\sigma_{a}^{2}\left(1+{\bar{F}}_{f,2000}-{\bar{\text{A}}}_{f,2000}\right)=90$. If estimated ${\hat{\sigma }}_{a\left(f\right)}^{2\left(2000\right)}$ is 75, the loss due to Bulmer effect and preselection is 90 – 75 = 15.

The genetic variance at equilibrium was calculated using the program SelAction 2.2 (Rutten et al., 2002), modeling a selection scheme similar to the actual one, based on progeny test for males, own phenotype for females, and parent average for young animals. We assumed 2 breeding objectives (milk yield alone or milk yield, composition, and SCS), corresponding to the change along the years of breeding objectives, and 3 heritabilities of 0.20, 0.25, and 0.30 around the estimated heritability of 0.28.

The estimated genetic variance at the base population for milk yield was (±SE, in liters squared, L^2^) 499.2 ± 5.2, permanent environmental variance was 409.3 ± 3.6, and residual variance was 857.3 ± 1.2. The estimated heritability (*h*^2^) was 0.28 ± 0.003. This *h*^2^ value is very similar to a previous estimate (Legarra et al., 2014).

In Figure 1, we present the evolution of overall relationship coefficient (twice the coancestry). There was a rapid increase in coancestry for AI males at the beginning of the breeding scheme, followed by a steady trend of about 0.002 increase per year. There is a steady increase in female-female and female-AI males coancestries, and trends for inbreeding are similar. Thus, a small reduction in the genetic variance resulting from drift is expected.

**Figure 1:**
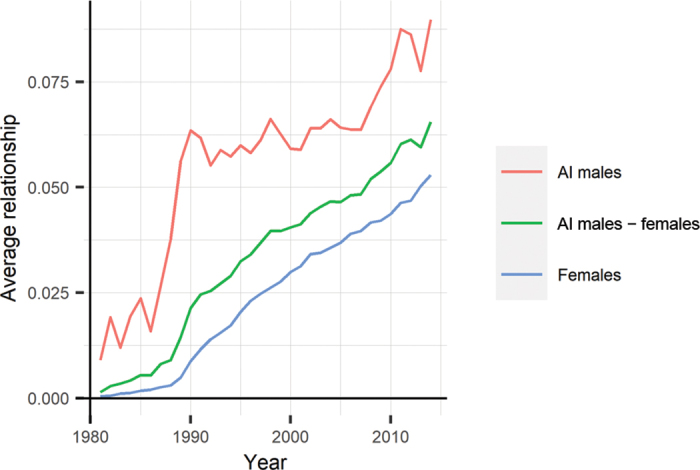
Evolution of average relationship per year of birth for AI males, females, and AI males − females.

In Figure 2, Figure 3 we present, for females and AI males, respectively, the genetic variance trajectory and the reduction of genetic variance due to drift and selection (Bulmer effect plus preselection at birth for AI males). For females, the lowest value of genetic variance was 438.6 ± 5.6 L^2^ in 2007 and, after 2011, the curve started to increase. For AI males, the genetic variance reached the lowest value (282.5 ± 19.9 L^2^) in 2008 and increased in the following years, as for females. The reduction in genetic variance for AI males was stronger than that for females. As mentioned previously, the trend for males included not only reduction due to drift and Bulmer effect, but also (and importantly), reduction due to strong preselection of males at birth based on parent average EBV. In contrast, in the female population, there is very little preselection pressure at birth.

**Figure 2:**
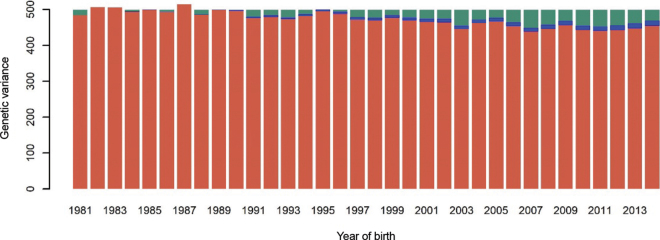
Partitioning of the genetic variance along the years for the female population. Red = observed genetic variance; blue = loss of genetic variance due to drift; green = loss of genetic variance due to selection.

**Figure 3:**
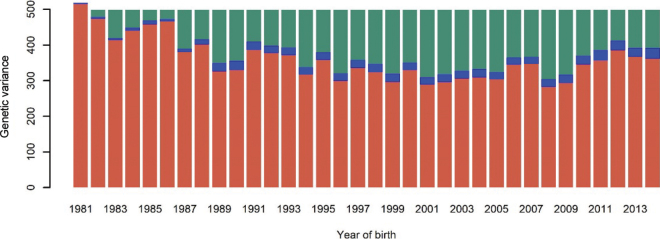
Partitioning of the genetic variance along the years for the AI male population. Red = observed genetic variance; blue = loss of genetic variance due to drift; green = loss of genetic variance due to selection and preselection at birth.

The increase in coancestry explains only a small proportion of the reduction of genetic variance in MTR, whereas the remaining was attributable to the Bulmer effect (in females) and to the Bulmer effect and selection at birth (in AI males). The reduction due to the Bulmer effect (and selection) seemed to stabilize in the last decade. However, it is not possible to say whether this plateau was due to stabilization of the Bulmer effect (as predicted by theory) or to changes in the breeding objective (as happens in this breeding scheme).

Finally, the results for the theoretical reduction in genetic variance computed with SelAction are as follows. A scheme selecting for milk yield (breeding objective at the beginning of the scheme) has, at equilibrium, a 15% reduction due to Bulmer effect, whereas a scheme selecting for milk yield, composition, and SCS has an 8% loss for milk yield. These numbers agree with our estimations of an approximate 10% loss due to the Bulmer effect in females. The cumulated loss due to increased coancestry was approximately 3% in both cases.

Our results have interest on their own. To our knowledge, there are few estimates of the reduction of genetic variance with real data (Allier et al., 2019; Hidalgo et al., 2020), and none of them disentangle the effect of drift from the effect of selection. What we observe is that the reduction due to drift is small, in spite of popular concerns regarding the increase of inbreeding. The reduction due to selection is larger, but it dissipates with the change in selection objectives. This suggests that a breeding scheme with mild control of effective population size, coupled with changes in breeding objectives, should be enough to avoid important loss of genetic variability.

In conclusion, for milk yield in MTR dairy sheep, there has been a steady reduction of genetic variance due to drift (roughly 3% in 30 yr) and reduction due to selection (roughly 10% in 30 yr). The loss due to selection reached an asymptotic value due either to the nature of the Bulmer effect or to the change of selection objectives. These results highlight that there is a need to check the evolution of genetic variability and that common strategies diversifying selection objectives and controlling inbreeding result in small losses of genetic variation.
